# Supplementary material for: Non-invasive MR imaging of human brain lymphatic networks with connections to cervical lymph nodes
Source: Nat Commun. 2022 Jan 11;13:203. doi: 10.1038/s41467-021-27887-0 (PMC8752739; doi:10.1038/s41467-021-27887-0)
Supplement: Supplementary file 1 — Supplementary Information [file 41467_2021_27887_MOESM1_ESM.pdf]

Supplementary Figures

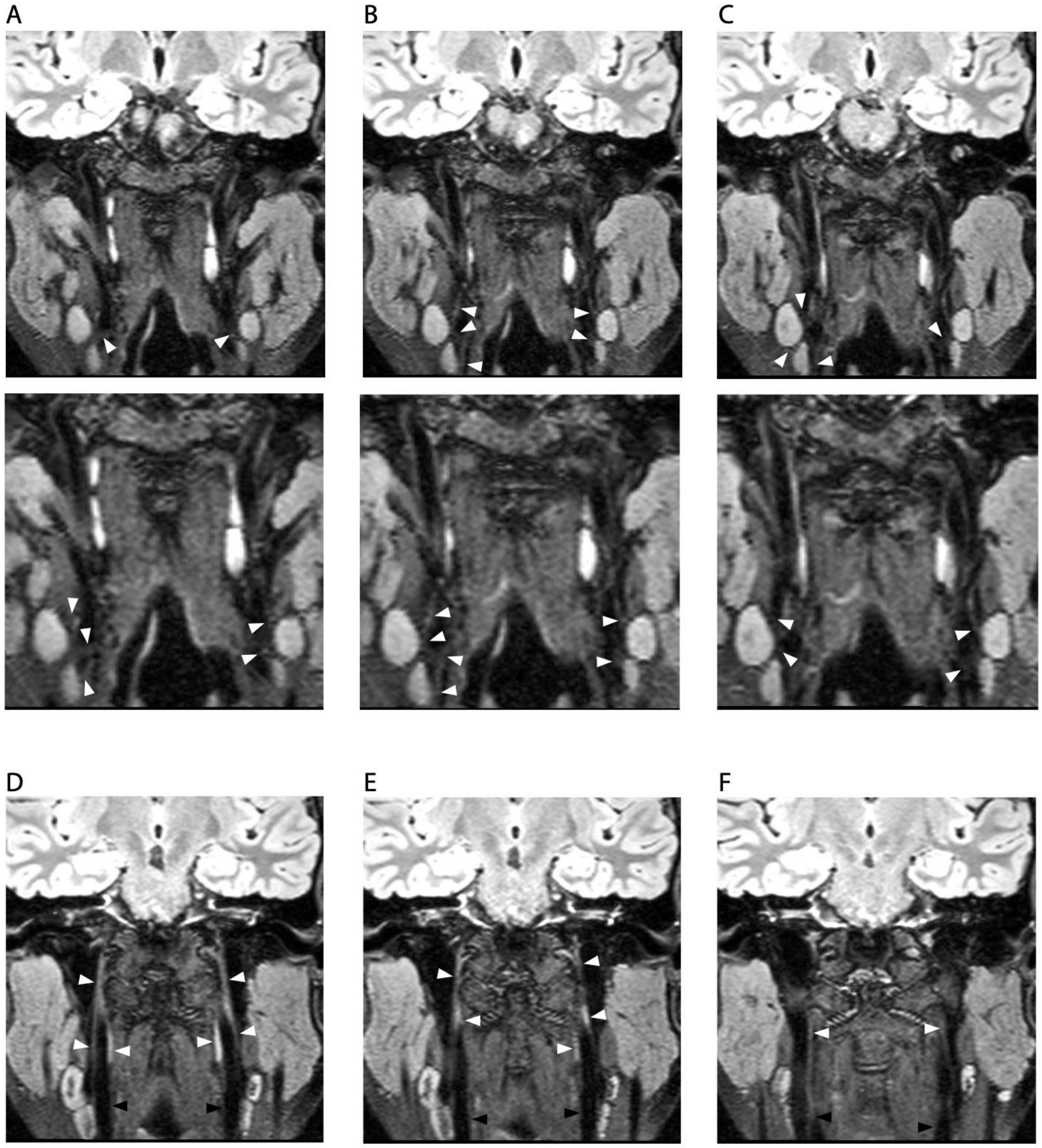

Supplementary Fig. 1. 22-year-old female subject. Sequential coronal FLAIR images depict connections between lymph nodes and structures in the vascular space including the wall of the internal carotid artery, the wall of the jugular vein, and the cervical cranial nerves (arrowheads). Additionally, coronal images (D-F) reveal attenuation of fluid drainage channels below the cervical lymph nodes along the vessels and cranial nerves (arrowheads).

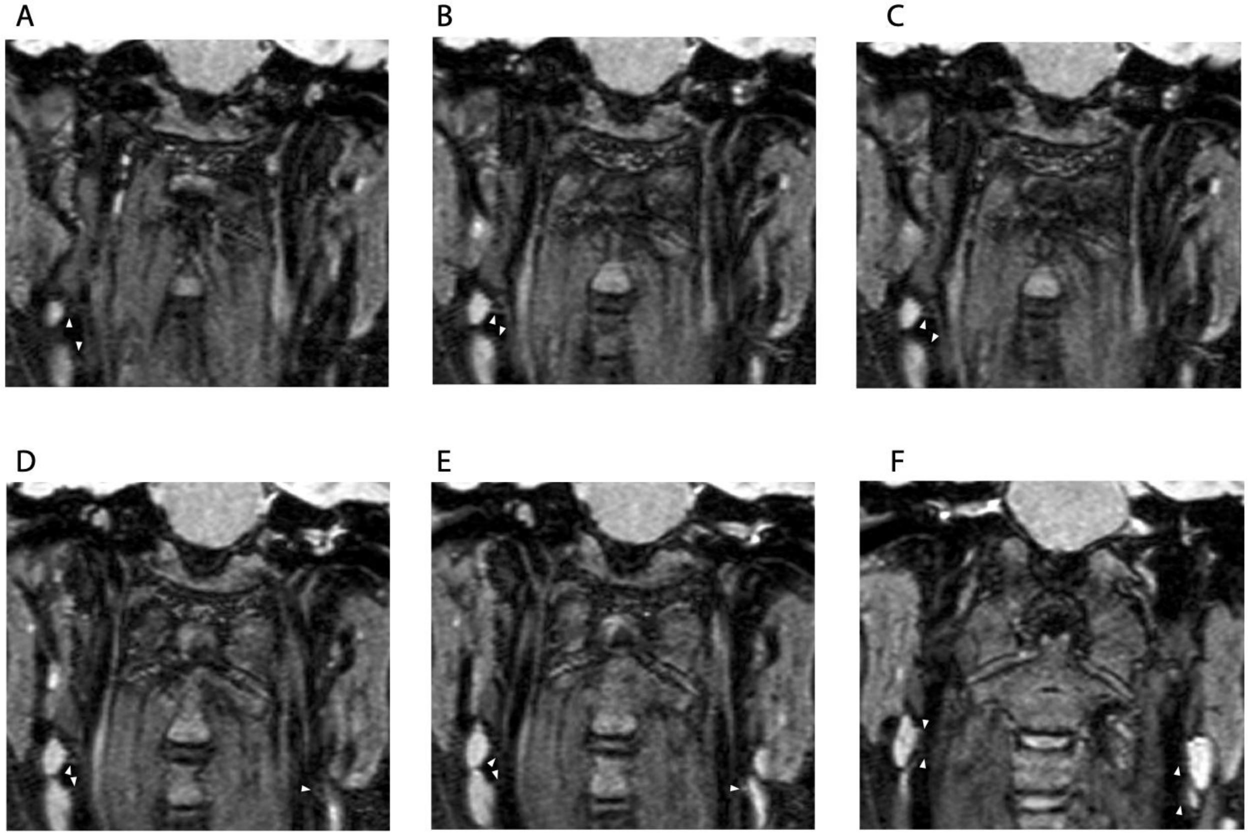

Supplementary Fig. 2. 17-year-old male subject. Sequential coronal FLAIR images depict connections between the lymph nodes and structures in the vascular space including the wall of the internal carotid artery, jugular vein and the cervical cranial nerves (arrowheads).

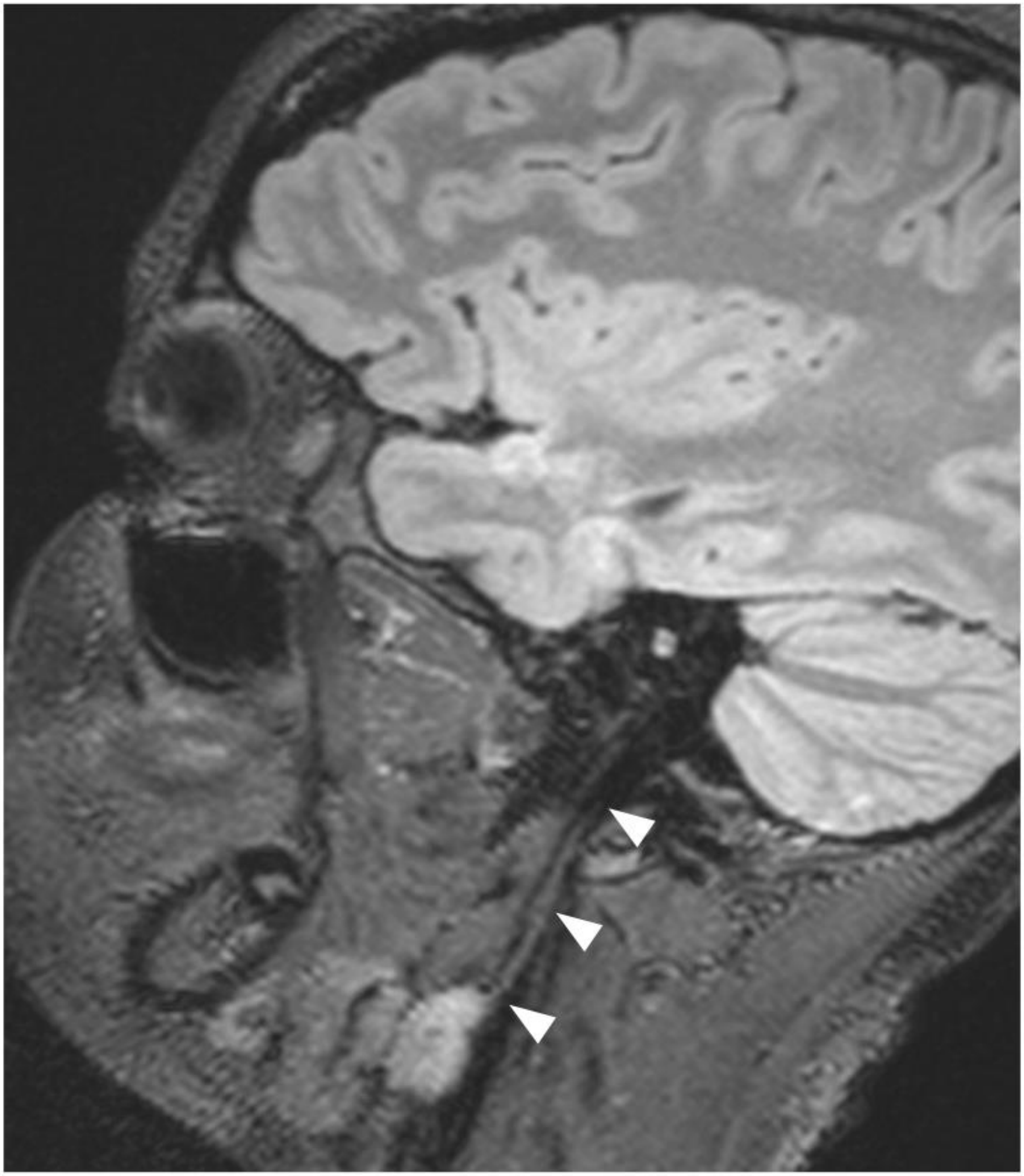

Supplementary Fig. 3. 20-year-old female subject. Sagittal oblique FLAIR images show direct communication between the cervical cranial nerves and vascular deep cervical lymph nodes (arrowheads).

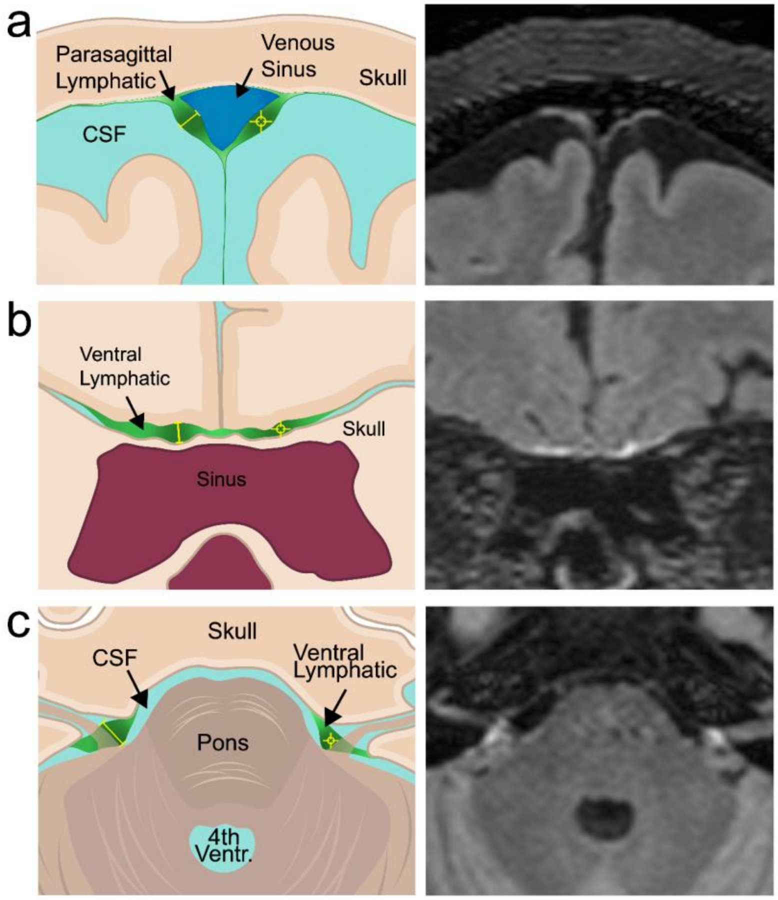

d

Dorsal Lymphatic Variables

Anterior Superior Sagittal Sinus
Middle Superior Sagittal Sinus
Sinus Rectus
Sinus Confluence
Right Sigmoid Sinus-Transverse Sinus Junctions
Left Sigmoid Sinus-Transverse Sinus Junctions

Ventral Lymphatic Variables

Anterior Cranial Fossa Dura
Optic Groove Dura
Right Meckel`s Orifice Dura
Left Meckel`s Orifice Dura
Right Meckel`s Dura
Left Meckel`s Dura
Right Dura at The Orifice of The Internal Auditory Canal
Left Dura at The Orifice of The Internal Auditory Canal
Right Dura at The Orifice of The Jugular Foramen
Left Dura at The Orifice of The Jugular Foramen

Supplementary Fig. 4. Schematic representation of our measurement technique. Corresponding MR images from the mid portion of the superior sagittal sinus (a), anterior cranial fossa dura (b), and dura at the level of the internal auditory canals (c). This list includes all dorsal and ventral variables that could be measured in all subjects (d).

Supplementary Tables

Phantom Number	Albumin Concentration (mg/dl)	TI 1400 SI (Mean ± SD)
1	ddH_2O	25.7 ± 3.4
2	7.5	27.6 ± 3.5
3	15	32.8 ± 4.8
4	30	22.7 ± 5.5
5	60	15.6 ± 4.8
6	120	16.6 ± 6.0
7	240	19.3 ± 2.7
8	500	32.2 ± 1.8
9	1000	50.0 ± 3.8
10	2000	76.2 ± 2.5
Dural Lymphatic Signal		103.1 ± 16.0
11	4000	105.8 ± 2.4

Supplementary Table 1. SI values measured from solutions of different albumin concentrations at TI 1400 ms.

Anatomical Structures	TI 1800<br>SI (Mean ± SD)
Carotid Artery	11.3 ± 2.3
Dural Venous Sinus	13.0 ± 1.6
Cerebrospinal Fluid	14.2 ± 2.1
Neck Muscles	64.6 ± 3.6
White Matter	97.3 ± 3.8
Grey Matter	109.4 ± 3.1
Dural Lymphatic	114.2 ± 6.0
Lymph Nodes	170.9 ± 10.3

Supplementary Table 2. Mean signal intensity measurements from key structures in the head and neck regions of healthy subjects.

	Signal Intensity (units)	Signal Intensity Min-Max (units)	Thickness (mm)	Thickness Min-Max (mm)
Sagittal Sinus Anterior	132±23	59–207	2.2±0.6	0.7–3.7
Sagittal Sinus Middle	158±23	97–201	3.9±1.1	2.5–7.0
Sagittal Sinus Posterior	98±31	24–180	1.5±0.5	0.9–4.1
Sinus Rectus	119±31	32–219	1.3±0.5	0.6–3.4
Confluence	164±43	41–281	2.8±1.4	0.9–7.2
Right Transverse Sinus	78±38	20–151	1.1±0.3	0.5–2.4
Left Transverse Sinus	82±34	24–163	1.1±0.2	0.6–1.6
Right Sigmoid Sinus–Transverse Sinus Junction	112±43	32–204	1.7±0.6	0.8–3.4
Left Sigmoid Sinus–Transverse Sinus Junction	115±38	40–198	1.6±0.4	0.9–2.9
Right Sigmoid Sinus	57±28	21–136	1.0±0.2	0.6–1.5
Left Sigmoid Sinus	66±30	23–153	1.0±0.2	0.5–1.8
Right Jugular Vein Area	79±30	22–157	1.2±0.3	0.7–1.9
Left Jugular Vein Area	92±39	34–205	1.3±0.3	0.8–2.3
Posterior Foramen Magnum	231±71	103–416	2.7±1.2	0.8–6.2

Supplementary Table 3. Signal Intensity and Thickness within divisions of the Dorsal Lymphatic System.

	Signal Intensity (units)	Signal Intensity Min-Max (units)	Thickness (mm)	Thickness Min-Max (mm)
Anterior Cranial Fossa Dura	245±39	158–343	1.4±0.4	0.7–2.7
Optic Groove Dura	277±53	113–388	2.1±0.8	1.0–4.5
Diaphragma Sellae	243±64	136–420	1.5±0.4	0.9–2.8
Right Trigeminal Dura Orifice	256±68	134–463	2.2±0.6	1.3–4.5
Left Trigeminal Dura Orifice	254±73	120–442	2.1±0.7	1.2–4.4
Right Meckel's Cave Dura	205±33	146–319	1.3±0.1	1.1–7.0
Left Meckel's Cave Dura	204±36	137–365	1.3±0.2	1.2–1.0
Right Internal Acoustic Canal Orifice Dura	301±60	173–433	2.6±0.8	1.4–5.5
Left Internal Acoustic Canal Orifice Dura	313±68	190–539	2.5±0.7	1.4–4.4
Right Jugular Foramen Dura	256±65	142–453	2.3±0.7	1.1–3.8
Left Jugular Foramen Dura	243±61	135–409	2.2±0.8	1.1–5.1

Supplementary Table 4. Signal Intensity and Thickness within divisions of the Ventral Lymphatic System.

	Older	Younger	p
Right ICA-JV-CN IX-XII (%visible)	100	100	1
Left ICA-JV-CN IX-XII (%visible)	100	100	1
Right ICA-JV-CN IX-XII RLG LN (%visible)	86.2	100	0.014*
Left ICA-JV-CN IX-XII RLG LN (%visible)	93.1	100	0.125*
Right ICA-JV-CN IX-XII-vascular LN (%visible)	96.6	100	0.358*
Left ICA-JV-CN IX-XII-vascular LN (%visible)	96.6	100	0.358*

Supplementary Table 5. Connections between anatomical structures and lymph nodes in the neck region. ICA: internal carotid artery, JV:Jugular vein, CN: cranial nerve, RLG: retropharyngeal lymph node, LN: lymph node

* Fisher test is used

	Older	Younger	Test*	95% CI	p
Sagittal anterior SI (mean [SD])	137 (23)	130 (23)	-1.39	-18.1 - 3.2	0.168
Sagittal anterior SI / muscle SI ratio (mean [SD])	1.8 (0.31)	1.7 (0.33)	-1.22	-0.24 - 0.06	0.23
Sagittal anterior thickness (mean [SD])	2.4 (0.5)	2.1 (0.6)	-2.55	-0.58 - -0.07	0.013
Sagittal middle SI (mean [SD])	166 (21)	154 (23)	-2.4	-22.4 - -2.1	0.019
Sagittal middle SI / muscle SI ratio (mean [SD])	2.2 (0.26)	2 (0.3)	-2.36	-0.28 - -0.024	0.021
Sagittal middle thickness (mean [SD])	4.5 (1.2)	3.6 (0.8)	-4.1	-1.37 - -0.47	<0.001
Sagittal posterior (%visible)	96.6	59.6	NA	NA	<0.001
Average sagittal SI (mean [SD])	151.4 (19.9)	141.5 (21.2)	-2.04	-19.4 - -0.25	0.044
Average sagittal SI / muscle SI ratio (mean [SD])	2 (0.26)	1.9 (0.28)	-1.89	-0.25 - 0.007	0.063
Average sagittal thickness (mean [SD])	3.4 (0.67)	2.8 (0.55)	-4.55	-0.9 - -0.35	<0.001
Transverse sinus visible (%)	82.8	50	8.46	NA	0.004
Sigmoid visible (%)	89.7	53.8	NA	NA	0.001
Jugular visible (%)	69	17.3	21.62	NA	<0.001

Average dorsal SI (mean [SD])	140.3 (21.1)	130.4 (23.6)	-1.87	-20.4 - 0.64	0.065
Average dorsal SI / muscle ratio (mean [SD])	1.8 (0.2)	1.7 (0.3)	-1.99	-0.24 - 0.001	0.05
Average ventral SI (mean [SD])	247.4 (35.4)	259.9 (32.6)	1.6	-3.03 - 28.03	0.11
Average ventral SI / muscle ratio (mean [SD])	3.3 (0.4)	3.5 (0.4)	1.93	-0.006 - 0.39	0.057
Dorsal/ventral SI ratio (mean [SD])	0.57 (0.08)	0.5 (0.07)	-3.97	-0.11 - -0.035	<0.001
Average dorsal thickness (median [IQR])	2.6 (0.8)	2.1 (0.5)	-4.39	NA	<0.001
Average ventral thickness (median [IQR])	2 (0.5)	1.9 (0.5)	-1.98	NA	0.048
Dorsal/ventral thickness ratio (median [IQR])	1.19 (0.34)	1.06 (0.33)	-3.09	NA	0.002
Average dorsal SI/thickness ratio (mean [SD])	55.2 (9.3)	63.7 (10.3)	3.67	3.9 - 13.1	<0.001
Average ventral SI/thickness ratio (mean [SD])	119.3 (17.3)	136.8 (22.7)	3.60	7.8 - 27.2	0.001
Average AVS SI (mean [SD])	248 (42)	259 (34)	1.26	-6.29 - 27.82	0.21
Average AVS SI / muscle SI ratio (mean [SD])	3.27 (0.47)	3.46 (0.55)	1.58	-0.05 - 0.43	0.119
Average AVS thickness (mean [SD])	1.9 (0.3)	1.5 (0.3)	-5.2	-0.53 - -0.24	<0.001

Average PVS SI (mean [SD])	258 (45)	277 (44)	1.88	-1.18 - 39.6	0.064
Average PVS SI / muscle SI ratio (mean [SD])	3.41 (0.55)	3.69 (0.56)	2.19	0.026 - 0.54	0.032
Average PVS thickness (mean [SD])	2.4 (0.5)	2.3 (0.5)	-0.83	-0.32 - 0.13	0.4
Retropharyngeal LN SI (mean [SD])	157 (24)	195 (28)	6.10	25.49 - 50.2	<0.001
Retropharyngeal LN SI / muscle SI ratio (mean [SD])	2.09 (0.34)	2.61 (0.46)	5.31	0.33 - 0.71	<0.001
Retropharyngeal LN short axis (mean [SD])	2.7 (1)	4.6 (1.5)	6.10	1.3 - 2.56	<0.001
Vascular LN SI (mean [SD])	150 (35)	184 (29)	4.75	19.73 - 48.23	<0.001
Vascular LN SI / muscle SI ratio (mean [SD])	1.99 (0.45)	2.47 (0.49)	4.38	0.26 - 0.70	<0.001
Vascular LN short axis (mean [SD])	5.9 (1.8)	7.6 (2.2)	3.56	0.76 - 2.70	<0.001

Supplementary Table 6. Comparison of older and younger subjects. SI: Signal intensity, LN: lymph node, AVS: anterior ventral system, PVS: posterior ventral system.

*Chi square, t, or Z values for categorical, skewly distributed continuous, or normally distributed continuous variables. All tests are two-sided.

NA: not available

	Males	Females	Test*	95% CI	p
Age (median [IQR])	31 (37.8)	43 (38.5)	-0.39	-10.9 - 7.36	0.701
Sagittal anterior SI (mean [SD])	141 (23)	125 (21)	3.42	6.95 - 26.34	0.001
Sagittal anterior SI / WM SI ratio (mean [SD])	1.14 (0.18)	0.99 (0.15)	3.91	0.07 - 0.22	<0.001
Sagittal anterior thickness (mean [SD])	2.3 (0.6)	2.1 (0.6)	2.0	0.001 - 0.50	0.049
Sagittal middle SI (mean [SD])	166 (18)	151 (24)	3.14	5.52 - 24.63	0.002
Sagittal middle SI / WM SI ratio (mean [SD])	1.34 (0.14)	1.20 (0.17)	3.87	0.07 - 0.21	<0.001
Sagittal middle thickness (mean [SD])	3.9 (0.9)	3.9 (1.2)	0.25	-0.42 - 0.54	0.8
Sagittal posterior (%visible)	77.8)	68.9	0.8	NA	0.371
Average sagittal SI (mean [SD])	153.9 (18.7)	138 (20.6)	3.59	7.06 - 24.66	0.001
Average sagittal SI / WM SI ratio (mean [SD])	1.24 (0.14)	1.10 (0.14)	4.49	0.08 - 0.21	<0.001
Average sagittal thickness (median [IQR])	3.08 (0.88)	2.9 (0.98)	-1.33	NA	0.185
Transverse sinus visible (%)	72.2	53.3	3.02	NA	0.082
Sigmoid visible (%)	72.2	62.2	0.9	NA	0.343

Jugular visible (%)	50	24.4	5.68	NA	0.017
Average dorsal SI (mean [SD])	145.4 (19.6)	124.6 (21.7)	4.45	11.47 - 30.07	<0.001
Average dorsal SI / WM SI ratio (mean [SD])	1.17 (0.16)	0.99 (0.13)	5.87	0.12 - 0.25	<0.001
Average ventral SI (mean [SD])	264 (29)	248.5 (36.4)	2.08	0.67 - 30.31	0.04
Average ventral SI / WM SI ratio (mean [SD])	2.13 (0.25)	1.97 (0.24)	2.83	0.05 - 0.27	0.006
Dorsal/ventral SI ratio (mean [SD])	0.56 (0.08)	0.5 (0.08)	2.86	0.016 - 0.087	0.005
Average dorsal thickness (median [IQR])	2.38 (0.8)	2.05 (0.6)	-3.18	NA	0.001
Average ventral thickness (median [IQR])	2.14 (0.52)	1.83 (0.35)	-3.22	NA	0.001
Dorsal/ventral thickness ratio (median [IQR])	1.17 (0.4)	1.09 (0.19)	-0.69	NA	0.492
Average dorsal SI/thickness ratio (mean [SD])	60.9 (10.8)	60.4 (10.8)	0.19	-4.37 - 5.29	0.85
Average ventral SI/thickness ratio (mean [SD])	126.4 (23.5)	133.9 (21.3)	-1.49	-17.38 - 2.47	0.139
Average AVS SI (mean [SD])	257 (31)	254 (42)	0.36	-13.61 - 19.59	0.721
Average AVS SI / WM SI ratio (mean [SD])	2.07 (0.25)	2.01 (0.27)	0.96	-0.058 - 0.17	0.328

Average AVS thickness (mean [SD])	1.8 (0.4)	1.6 (0.4)	2.21	0.18 - 0.34	0.03
Average PVS SI (mean [SD])	282 (41)	261 (46)	2.08	0.9 - 40.08	0.041
Average PVS SI / WM SI ratio (mean [SD])	2.28 (0.35)	2.08 (0.33)	2.6	0.05 - 0.35	0.011
Average PVS thickness (mean [SD])	2.5 (0.6)	2.2 (0.3)	3.21	0.13 - 0.56	0.002
Retropharyngeal LN SI (mean [SD])	184 (35)	180 (30)	0.65	-9.74 - 19.09	0.521
Retropharyngeal LN SI / WM SI ratio (mean [SD])	1.48 (0.27)	1.43 (0.23)	0.96	-0.06 - 0.16	0.342
Retropharyngeal LN short axis (mean [SD])	4 (1.8)	3.9 (1.5)	0.27	-0.64 - 0.84	0.786
Vascular LN SI (mean [SD])	173 (39)	172 (32)	0.14	-14.46 - 16.71	0.886
Vascular LN SI / WM SI ratio (mean [SD])	1.39 (0.31)	1.37 (0.26)	0.38	-0.1 - 0.15	0.704
Vascular LN short axis (mean [SD])	7.1 (2.4)	6.9 (2.2)	0.23	-0.89 - 1.12	0.820

Supplementary Table 7. Comparison of male and female subjects. SI: Signal intensity, WM: central white matter, AVS: anterior ventral system, PVS: posterior ventral system, LN: lymph node.

*Chi square, t, or Z values for categorical, skewly distributed continuous, or normally distributed continuous variables. All tests are two-sided. NA: not available

	Older subjects	Younger subjects	t*	95% CI	p
Right superficial temporalis muscle	76.3 (SD 8.5)	75.8 (SD 9.8)	-0.27	-4.9 - 3.72	0.78
Left superficial temporalis muscle	75.6 (SD 8.3)	75.7 (SD 9.6)	0.07	-4.09 - 4.37	0.95
Right insula	181.6 (SD 18.9)	191.8 (SD 20.6)	2.20	0.98 - 19.43	0.031
Left insula	180.9 (SD 19.2)	190.2 (SD 19.6)	2.07	0.36 - 18.30	0.042
Right nucleus caudatus	155.8 (SD 15.3)	168.8 (SD 17.4)	3.35	5.27 - 20.68	0.001
Left nucleus caudatus	156 (SD 15.4)	168 (SD 17.8)	3.04	4.14 - 19.79	0.003
Right cerebellum cortex	192.7 (SD 19.1)	200.6 (SD 21.1)	1.67	-1.52 - 17.33	0.10
Left cerebellum cortex	192.7 (SD 17.5)	201.5 (SD 20.7)	1.93	-0.21 - 17.91	0.06
Right cerebellum white matter	145.6 (SD 13.2)	147 (SD 16.5)	0.41	-5.62 - 8.56	0.68
Left cerebellum white matter	145.1 (SD 13.6)	147.8 (SD 15.9)	0.77	-4.3 - 9.67	0.45
Right frontal cortex	130.3 (SD13.5)	132.7 (SD 13.1)	0.79	-3.69 - 8.56	0.43
Left frontal cortex	130.9 (SD 13.4)	132.1 (SD 12.9)	0.39	-5 - 7.4	0.70
Right central white matter	125.3 (SD 11.9)	125.6 (SD 12.7)	0.086	-5.49 - 5.98	0.932
Left central white matter	125.3 (SD 10.8)	125.3 (SD 12.6)	0.07	-5.5 - 5.55	0.994

Supplementary Table 8. Signal intensity of reference areas and their association with age.

*All tests are two-sided

	Male subjects	Female subjects	t*	95% CI	p
Right superficial temporalis muscle	78.4 (SD 7.6)	74 (SD 10.1)	2.17	0.37 - 8.46	0.033
Left superficial temporalis muscle	78.3 (SD 7.3)	73.5 (SD 9.9)	2.4	0.8 - 8.69	0.019
Right insula	192.8 (SD 21.0)	184.5 (SD 19.5)	1.85	-0.64 - 17.32	0.068
Left insula	191.4 (SD 19.5)	183.2 (SD 19.6)	1.87	-0.53 - 16.86	0.065
Right nucleus caudatus	167.7 (SD 16.8)	161.2 (SD 18.1)	1.65	-1.33 - 14.29	0.10
Left nucleus caudatus	167.4 (SD 16.4)	160.7 (SD 18.5)	1.68	-1.21 - 14.47	0.10
Right cerebellum cortex	205.1 (SD 18.3)	191.9 (SD 20.7)	2.98	4.35 - 21.9	0.004
Left cerebellum cortex	205.4 (SD 18.3)	192.7 (SD 19.7)	2.97	4.2 - 21.17	0.004
Right cerebellum white matter	149.6 (SD 16.7)	144 (SD 13.8)	1.68	-1.07 - 12.39	0.10
Left cerebellum white matter	149.6 (SD 15.8)	144.6 (SD 14.3)	1.47	-1.76 - 11.58	0.15
Right frontal cortex	132.5 (SD 12.8)	131.3 (SD 13.7)	0.4	-4.74 - 7.11	0.69
Left frontal cortex	132.3 (SD 12.4)	131.2 (SD 14.2)	0.34	-4.95 - 7.02	0.73
Right central white matter	124.6 (SD 9.9)	126.2 (SD 14.1)	-0.58	-7.12 - 3.93	0.55
Left central white matter	124.2 (SD 9.9)	126.3 (SD 13.3)	-0.8	-7.46 - 3.17	0.42

Supplementary Table 9. Signal intensity of reference areas and their association with sex.

*All tests are two-sided.
