# Supplementary material for: Non-invasive MR imaging of human brain lymphatic networks with connections to cervical lymph nodes
Source: Nat Commun. 2022 Jan 11;13:203. doi: 10.1038/s41467-021-27887-0 (PMC8752739; doi:10.1038/s41467-021-27887-0)
Supplement: Supplementary file 3 — Reporting Summary [file 41467_2021_27887_MOESM3_ESM.pdf]

nature research

Corresponding author(s): Mehmet Sait Albayram

Last updated by author(s): Nov 17, 2021

Reporting Summary

Nature Research wishes to improve the reproducibility of the work that we publish. This form provides structure for consistency and transparency in reporting. For further information on Nature Research policies, see our Editorial Policies and the Editorial Policy Checklist.

Statistics

For all statistical analyses, confirm that the following items are present in the figure legend, table legend, main text, or Methods section.

n/a	Confirmed	
☐	☒	The exact sample size (*n*) for each experimental group/condition, given as a discrete number and unit of measurement
☐	☒	A statement on whether measurements were taken from distinct samples or whether the same sample was measured repeatedly
☐	☒	The statistical test(s) used AND whether they are one- or two-sided<br>*Only common tests should be described solely by name; describe more complex techniques in the Methods section.*
☐	☒	A description of all covariates tested
☐	☒	A description of any assumptions or corrections, such as tests of normality and adjustment for multiple comparisons
☐	☒	A full description of the statistical parameters including central tendency (e.g. means) or other basic estimates (e.g. regression coefficient) AND variation (e.g. standard deviation) or associated estimates of uncertainty (e.g. confidence intervals)
☐	☒	For null hypothesis testing, the test statistic (e.g. *F*, *t*, *r*) with confidence intervals, effect sizes, degrees of freedom and *P* value noted<br>*Give P values as exact values whenever suitable.*
☒	☐	For Bayesian analysis, information on the choice of priors and Markov chain Monte Carlo settings
☒	☐	For hierarchical and complex designs, identification of the appropriate level for tests and full reporting of outcomes
☐	☒	Estimates of effect sizes (e.g. Cohen's *d*, Pearson's *r*), indicating how they were calculated

Our web collection on statistics for biologists contains articles on many of the points above.

Software and code

Policy information about availability of computer code

Data collection	Performed in the hospital Picture Archiving and Communication Systems (PACS): Visage Visage 7.1.14 (Visage Imaging, Australia), The volBrain Pipeline (volBrain version 1.0 release 04/03/2015) (https://www.volbrain.upv.es)
Data analysis	SPSS 20·0 (IBM, Armonk, New York)

For manuscripts utilizing custom algorithms or software that are central to the research but not yet described in published literature, software must be made available to editors and reviewers. We strongly encourage code deposition in a community repository (e.g. GitHub). See the Nature Research guidelines for submitting code & software for further information.

Data

Policy information about availability of data

All manuscripts must include a data availability statement. This statement should provide the following information, where applicable:
- Accession codes, unique identifiers, or web links for publicly available datasets
- A list of figures that have associated raw data
- A description of any restrictions on data availability

The minimum dataset necessary to interpret, verify and extend the research in this article is accessible within the manuscript and its supplementary information. The source data available in the Supplementary material, anonymized images, and additional data in this work are available on request. The authors have access to all data used in the study.

Field-specific reporting

Please select the one below that is the best fit for your research. If you are not sure, read the appropriate sections before making your selection.

☒ Life sciences ☐ Behavioural & social sciences ☐ Ecological, evolutionary & environmental sciences

For a reference copy of the document with all sections, see nature.com/documents/nr-reporting-summary-flat.pdf

Life sciences study design

All studies must disclose on these points even when the disclosure is negative.

Sample size	No sample-size calculation was performed. the sample size 81. All eligible cases are included.
Data exclusions	Patients were excluded from the study if the quality of imaging was insufficient to recognize the dural and subarachnoid spaces, if significant motion artifacts were present, if contrast administration occurred within a few weeks before 3D FLAIR series collection, or if imaging was performed under sedation or general anesthesia. Patients with previous surgery, additional diagnoses such as stroke, intracranial mass, multiple sclerosis, focal parenchymal disease, significant atrophy, significant small vessel disease, or congenital developmental abnormalities were excluded.
Replication	As this is a retrospective study, replication with the same patients is not possible. The phantom studies were carried out multiple times. The same imaging findings that we obtained can be obtained with our study MR parameters and protocols at any time or place. When we use the same MR parameters for brain MR imaging, we can detect dorsal and ventral lymphatic signals in different patients in our current clinical setting. For replication of the measurements, we assessed intra-observer variability to evaluate possibility for replication. S.A. made measurements in 10 of the patients on two different times and without seeing his previous measurements. However, it was not feasible to assess inter-observer variability because of the large work load.
Randomization	Observational study design. The randomization was not done.
Blinding	We evaluated anatomical structures including SI and thickness measurements. The measurements were performed on MR images on which age, gender and other identifying patient attributes were blinded.

Reporting for specific materials, systems and methods

We require information from authors about some types of materials, experimental systems and methods used in many studies. Here, indicate whether each material, system or method listed is relevant to your study. If you are not sure if a list item applies to your research, read the appropriate section before selecting a response.

Materials & experimental systems

n/a	Involved in the study	
☒	☐	Antibodies
☒	☐	Eukaryotic cell lines
☒	☐	Palaeontology and archaeology
☒	☐	Animals and other organisms
☐	☒	Human research participants
☒	☐	Clinical data
☒	☐	Dual use research of concern

Methods

n/a	Involved in the study	
☒	☐	ChIP-seq
☒	☐	Flow cytometry
☐	☒	MRI-based neuroimaging

Human research participants

Policy information about studies involving human research participants

Population characteristics	We investigated patients referred for MR imaging due to clinical history of epilepsy or suspicion of epilepsy. A total of 81 subjects (45 females and 36 males) with a mean age of 41·7 (SD 20·4, range 15-80) years were included.
Recruitment	The subjects were selected from those who underwent MR imaging for clinical history of epilepsy or suspicion of epilepsy during a consecutive 13 month period. This is a retrospective study. We included all eligible subjects. We identify no selection biases. Exclusion criteria are given in the data exclusions section.
Ethics oversight	The study is registered in IRB- IRB201902528.

Note that full information on the approval of the study protocol must also be provided in the manuscript.

Magnetic resonance imaging

Experimental design

Design type	This was not fMRI study. Neither resting state or task related block design done.
Design specifications	This was not fMRI study.
Behavioral performance measures	This was not fMRI study.

Acquisition

Imaging type(s)	3D fluid-attenuated inversion recovery (FLAIR) series without contrast injection
Field strength	3T Prisma scanner
Sequence & imaging parameters	All MR imaging was performed on a 3 T Prisma scanner (Siemens, Erlangen) with a multichannel head coil during a consecutive 13 month period. For each patient, images were obtained from whole-brain T2-FLAIR scan with fat saturation (Sagittal 3D acquisition, SPACE sequence, field-of-view 230x230, matrix 512x512, 0·9 mm sections, TR/TE/TI = 5000/387/1800 ms, nonselective inversion pulse, echo-train length 246, bandwidth 750Hz/pixel, acceleration factor 1, acquisition time 5 min, 42 seconds, with fat saturation). For Volumetric analysis, 3D-T1-weighted gradient-echo (MPRAGE-magnetization-prepared rapid acquisition of gradient echo, acquisition matrix 256 × 256, isotropic resolution 0.9 mm, 192 slices, repetition time [TR]/echo time [TE]/inversion time [TI]=1720/2.11/865 ms, flip angle 9, acquisition time 5 min 45 s).
Area of acquisition	Whole brain-skull and upper neck.

Diffusion MRI ☐ Used ☒ Not used

Preprocessing

Preprocessing software	No preprocessing performed, MR images directly analyzed in our PACS.
Normalization	MRI signal units measured at areas of interest were normalized to the reference tissues. As reference tisssues, SI values of bilateral insula, caudate nucleus, bilateral superficial temporalis muscle at the level of frontal skull base, bilateral precentral frontal cortex, bilateral posterior centrum semiovale, bilateral central cerebellar white matter, and cortex at the level of the dentate nucleus were measured.
Normalization template	No template used.
Noise and artifact removal	Not used.
Volume censoring	Not used.

Statistical modeling & inference

Model type and settings	Differences between continuous data were analyzed using univariate models. Correlations were tested using Spearman's correlation coefficient. Statistical significance was set at the 0.05 level (two-tailed).
Effect(s) tested	We studied signal intensity and thickness by ROI measurement of normalized and standard MRI signal. We also assessed the visibility of specific anatomical structures.

Specify type of analysis: ☐ Whole brain ☐ ROI-based ☒ Both

Anatomical location(s)	Dorsal and Ventral anatomical structures were selected by visual inspection and assessed in part by ROI and thickness analyses. The atlases and algorithms were not used. The whole brain volumetric analysis were done.

Statistic type for inference (See Eklund et al. 2016)	Not relevant.
Correction	Multiple comparisons were not performed.

Models & analysis

n/a	Involved in the study
☒	☐ Functional and/or effective connectivity
☒	☐ Graph analysis
☒	☐ Multivariate modeling or predictive analysis
